# Sabinene Prevents Skeletal Muscle Atrophy by Inhibiting the MAPK–MuRF-1 Pathway in Rats

**DOI:** 10.3390/ijms20194955

**Published:** 2019-10-08

**Authors:** Yunkyoung Ryu, Donghyen Lee, Seung Hyo Jung, Kyung-Jin Lee, Hengzhe Jin, Su Jung Kim, Hwan Myung Lee, Bokyung Kim, Kyung-Jong Won

**Affiliations:** 1 Department of Physiology, School of Medicine, Konkuk University, 120 Neungdong-ro, Gwangjin-gu, Seoul 05029, Korea; ykryu33@daum.net (Y.R.); bkkim2@kku.ac.kr (B.K.); 2 Department of Cosmetic Science, College of Life and Health Sciences, Hoseo University, 20 Hoseo-ro79beon-gil, Hoseo-ro, Baebang-eup, Asan 31499, Korea

**Keywords:** *Chrysanthemum boreale* Makino essential oil, sabinene, skeletal muscle atrophy, L6 cells, MAPK/MuRF-1

## Abstract

*Chrysanthemum boreale* Makino essential oil (CBMEO) has diverse biological activities including a skin regenerating effect. However, its role in muscle atrophy remains unknown. This study explored the effects of CBMEO and its active ingredients on skeletal muscle atrophy using in vitro and in vivo models of muscle atrophy. CBMEO reversed the size decrease of L6 myoblasts under starvation. Among the eight monoterpene compounds of CBMEO without cytotoxicity for L6 cells, sabinene induced predominant recovery of reductions of myotube diameters under starvation. Sabinene diminished the elevated E3 ubiquitin ligase muscle ring-finger protein-1 (MuRF-1) expression and p38 mitogen-activated protein kinase (MAPK) and extracellular signal-regulated kinase1/2 (ERK1/2) phosphorylations in starved myotubes. Moreover, sabinene decreased the increased level of reactive oxygen species (ROS) in myotubes under starvation. The ROS inhibitor antagonized expression of MuRF-1 and phosphorylation of MAPKs, which were elevated in starved myotubes. In addition, levels of muscle fiber atrophy and MuRF-1 expression in gastrocnemius from fasted rats were reduced after administration of sabinene. These findings demonstrate that sabinene, a bioactive component from CBMEO, may attenuate skeletal muscle atrophy by regulating the activation mechanism of ROS-mediated MAPK/MuRF-1 pathways in starved myotubes, probably leading to the reverse of reduced muscle fiber size in fasted rats.

## 1. Introduction

Diverse physiological and pathological conditions such as starvation, inactivity, aging, diabetes, and cancer can cause decreased synthesis and increased breakdown of muscle proteins, leading to decreased muscle tissues, known as muscle atrophy [1,2,3]. Muscle atrophy is characterized by reduction in muscle fiber’s cross-section area and protein contents, loss of muscle mass, and muscle weakness or a decrease in the ability to generate force, resulting in a skeletal muscle dysfunction [1,4]. Skeletal muscle atrophy can be prevented by therapies associated with appropriate diet and exercise [5]. However, a complete treatment for muscle atrophy has not been developed yet and is being actively researched. Therefore, it is necessary to develop more effective agents to overcome muscle atrophy. 

The ubiquitin–proteasome system is the major regulatory mechanism of muscle protein breakdown associated with skeletal muscle atrophy [6,7]. The muscle-specific E3 ubiquitin ligases, muscle atrophy F-Box (MAFbx) and muscle ring-finger-1 (MuRF-1), play important roles in ubiquitin-mediated protein degradation involved in skeletal muscle atrophy [8]. Levels of MuRF-1 and MAFbx are upregulated in skeletal muscles under various atrophy conditions, including starvation, inactivity, aging, diabetes, and cancer in animals and/or humans [9,10]. Mitogen-activated protein kinases (MAPKs), including extracellular signal-regulated kinase (ERK) 1/2, stress-activated protein kinase/c-Jun N-terminal kinase (JNK), and p38 MAPK, are signaling molecules that participate in skeletal muscle atrophy [11,12]. The p38 MAPK/nuclear factor-κB pathway regulates expression levels of MuRF-1 and MAFbx in muscles under oxidative stress that produces inflammatory cytokines such as tumor necrosis factor-alpha and interleukin-1 [13,14,15]. It is known that reactive oxygen species (ROS) as oxidative stressors can up-regulate MuRF-1 and MAFbx expression and lead to increased muscle protein degradation and myotube atrophy [16,17,18]. ROS inhibition by antioxidant protein can decrease MuRF-1 and MAFbx expression by inhibiting p38 MAPK activation in starved myoblasts [12,13]. Therefore, starvation-induced ROS may affect expression of E3 ubiquitin ligase via the MAPK pathway [11]. 

*Chrysanthemum boreale* Makino (CBM) is widely distributed in Asia, including Korea, China, and Japan. It has been used to treat various diseases due to its many biological activities, such as anti-tumor, anti-inflammation, anti-angiogenesis, and anti-hypertension effects [19,20]. Essential oils (EOs) isolated from various natural sources have been used as materials of aromatic and flavoring chemicals in food, industrial, and pharmaceutical products [21]. The EO of CBM (CBMEO) exhibits a variety of biological and pharmacological activities, including antioxidant, anti-melanogenic, and skin-regenerating effects [20,22]. In our previous study, we found that CBMEO contains 33 single components that might have different bioactivities [22]. However, whether CBMEO and its components can affect muscle atrophy is currently unclear. 

Therefore, in this study, we analyzed the effects of CBMEO and its components, especially monoterpenoids including sabinene, on myoblast atrophy. Based on results of this analysis, we explored the effect of sabinene on atrophy in starved myotubes, as well as its possible action mechanism. Furthermore, we investigated the in vivo effect of sabinene on muscle atrophy using a fasted animal model.

## 2. Results

### 2.1. Effect of CBMEO on Starvation-Induced Diminution of L6 Myoblast Cell Size

**Figure 1:**
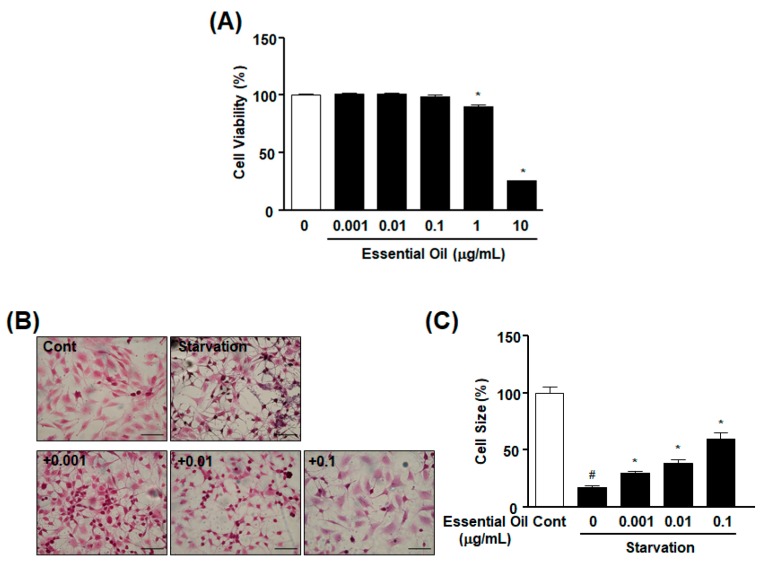
Effect of *Chrysanthemum boreale* Makino essential oil on starvation-induced atrophy of L6 cells. (**A**) Effect of *Chrysanthemum boreale* Makino essential oil (CBMEO) on L6 myoblast viability. Cells were incubated in the presence or absence of essential oil (0.001–10 μg/mL) for 48 h, and cell viability was measured using an XTT assay. Cellular response in the quiescent state was considered as 100% (*n* = 16). * *p* < 0.05 vs. untreated cells. (**B**,**C**) Effect of CBMEO on starvation-induced atrophy of L6 myoblasts. L6 myoblasts were incubated with serum-free Dulbecco’s modified Eagle’s medium (DMEM) in the presence or absence of essential oil (0.001–0.1 μg/mL) for 18 h and stained with crystal-violet to measure cell sizes. Cell sizes were measured as described in Materials and Methods. (**B**) Representative images. (**C**) Statistical data obtained from upper panel (**B**). Cell size in the quiescent state was considered as 100% (*n* = 9). Scale bar: 100 μm. * *p* < 0.05 vs. starved myoblasts in the absence of essential oil. # *p* < 0.05 vs. the quiescent state Cont, the quiescent state control without starvation.

To determine whether CBMEO affects L6 myoblast cell size, we first examined the effect of CBMEO on viability of L6 myoblasts using a 2,3-bis (2-methoxy-4-nitro-5-sulfophenyl)-2H-tetrazolium-5-carboxanilide inner salt (XTT) assay. Treatment with CBMEO did not affect the viability of L6 myoblasts at concentrations of 0.001 to 0.1 μg/mL. However, at concentrations of 1 and 10 μg/mL, CBMEO decreased the viability of L6 myoblasts (Figure 1A). These cell viability results allowed us to use CBMEO in a range of concentrations from 0.001 to 0.1 μg/mL in further experiments. We also tested the effect of CBMEO on cell sizes of L6 myoblasts that were decreased by incubation in low-glucose Dulbecco’s modified Eagle’s medium (DMEM) without fetal bovine serum (FBS) for 18 h. Treatment with CBMEO at concentrations of 0.001–0.1 μg/mL resulted in a dose-dependent reversal of the decrease in L6 cell size in response to starvation, showing the maximum effect at 0.1 μg/mL (Figure 1B,C).

### 2.2. Effects of Bioactive Components from CBMEO on Starvation-Decreased L6 Myoblast Size

**Figure 2:**
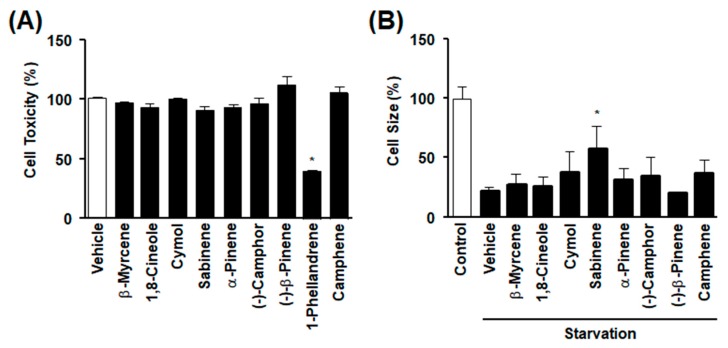
Effects of components identified from *Chrysanthemum boreale* Makino essential oil on starvation-induced atrophy of L6 cells. (**A**) Effects of components identified from CBMEO on L6 myoblasts’ viabilities. Cells were incubated in the presence or absence of nine components of monoterpenoids identified from CBMEO (300 μM for each component) for 48 h, and cell viability was measured using an XTT assay. Cellular response in the quiescent state was considered as 100% (*n* = 16). * *p* < 0.05 vs. untreated cells. (**B**) Effects of CBMEO components on starvation-induced atrophy of L6 myoblasts. L6 myoblasts were incubated with serum-free DMEM with or without a component identified from CBMEO for 18 h. Cell size in the quiescent state was considered as 100% (*n* = 12). * *p* < 0.05 vs. starved myoblasts in the absence of a component.

To identify the key biologically active ingredients involved in the restorative effect of CBMEO on muscle cell atrophy, we selected nine monoterpenoids that showed a peak area range of more than 1% among 33 components identified from CBMEO in a previous study [22]. We first tested effects of compounds on viabilities of L6 myoblasts using an XTT assay. Of these nine components of CBMEO, eight (except 1-phellandrene) failed to significantly affect viabilities of L6 myoblasts after treatment at a concentration of 300 μM (Figure 2A). We next examined whether these eight components that showed non-cytotoxicity affected starvation-induced L6 myoblast atrophy. Of these eight compounds, only sabinene recovered more than 50% of starvation-induced myoblast atrophy at a concentration of 300 μM (Figure 2B). These results led us to select sabinene as a major bioactive component of CBMEO that was capable of recovering atrophy of muscle cells induced by starvation.

### 2.3. Effect of Sabinene on Starvation-Induced Atrophy in Myotubes

**Figure 3:**
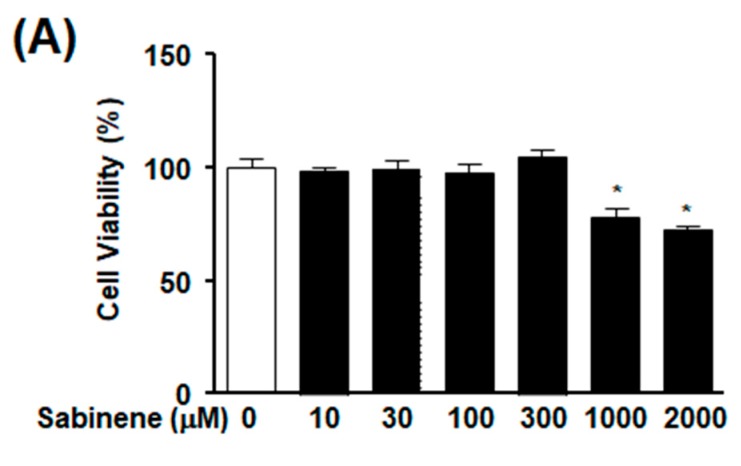

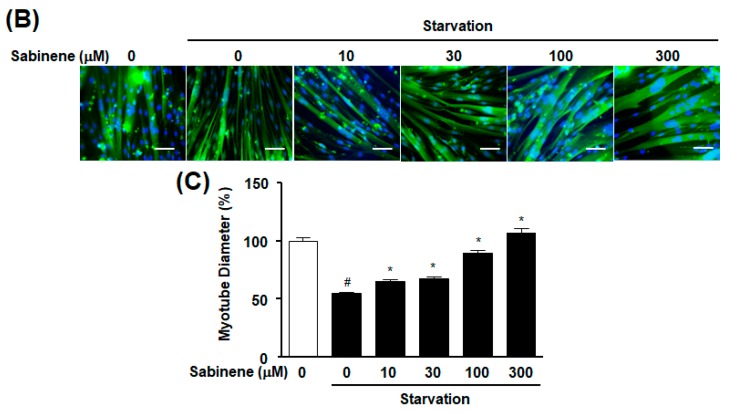
Effect of sabinene on atrophy of starved myotubes. (**A**) Effect of sabinene on myotube viability. L6 myotubes were incubated in the presence or absence of sabinene (10–2000 μM) for 48 h, and cell viability was measured using an XTT assay. Cell response in the quiescent state was considered as 100% (*n* = 16). * *p* < 0.05 vs. untreated cells. (**B**,**C**) Effect of sabinene on starvation-induced atrophy of L6 myotubes. L6 myotubes were incubated with serum-free DMEM in the absence or presence of sabinene (10–300 μM) for 18 h and immunostained with anti-myosin heavy-chain (MYH)-2 and anti-DAPI antibodies. Diameters of myotubes were measured as described in Materials and Methods. Green and blue indicate MYH-2-positive myotubes and DAPI-positive nucleus, respectively. Scale bar: 100 μm. (**B**) Representative images. (**C**) Statistical graph obtained from panel (**B**). Myotube size in the quiescent state was considered as 100% (*n* = 12). * *p* < 0.05 vs. starved myotubes without sabinene treatment. # *p* < 0.05 vs. the quiescent state.

To determine whether sabinene affects skeletal muscle cell atrophy, we tested effects of different concentrations of sabinene on muscle cells’ viabilities using an XTT assay. Treatment with sabinene did not affect the viability of differentiated L6 myotubes at 10–300 μM, although it did inhibit their viability at 1000 and 2000 μM (Figure 3A). We next tested the effect of sabinene on starvation-induced muscle cell atrophy by measuring myotube diameter. Treatment with sabinene at 10–300 μM inhibited starvation-induced decrease of myotube diameter in a dose-dependent manner (Figure 3B,C). The inhibitory effect of sabinene on starvation-induced decrease of myotube diameter was maximal at 300 μM. The diameter was about two times greater than that in the absence of sabinene under starvation-induced conditions (Figure 3C).

### 2.4. Effect of Sabinene on Atrophy-Related Signals in Myotubes

**Figure 4:**
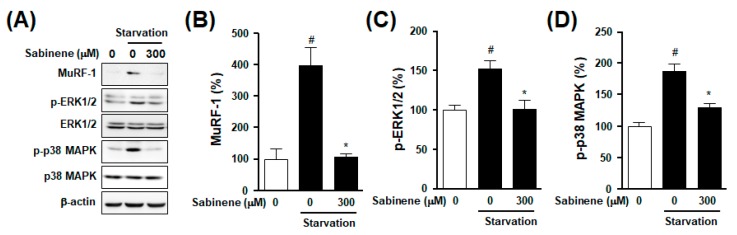
Effects of sabinene on muscle atrophy-related signaling proteins in starved myotubes. (**A**) Effects of sabinene on muscle atrophy-related signaling proteins. L6 myotubes were incubated with serum-free DMEM in the absence or presence of sabinene (300 μM) for 18 h. Myotube lysates were immunoblotted with indicated antibodies. (**B**–**D**) Statistical graphs obtained from panel (A). Expression of each protein in the quiescent state was considered as 100% (*n* = 10 for each protein). * *p* < 0.05 vs. starved myotubes in the absence of sabinene. # *p* < 0.05 vs. the quiescent state. p-ERK1/2, phosphorylated ERK1/2; p-p38 MAPK, phosphorylated p38 MAPK.

MuRF-1 and MAPKs are known as important signals that participate in skeletal muscle atrophy and starvation-induced muscle cell atrophy [12,17,23]. To determine the mechanism involved in the inhibitory effect of sabinene on starvation-induced atrophy in myotubes, myotubes were starved in the presence or absence of sabinene. MuRF-1 expression and MAPK phosphorylation were then analyzed using an immnunoblotting technique. As shown in Figure 4A,B, starved myotubes showed increased expression in MuRF-1. However, such increase was decreased by treatment with 300 μM sabinene. In addition, phosphorylation levels of ERK1/2 and p38 MAPK were enhanced in starved myotubes, whereas these levels were attenuated by treatment with sabinene (300 μM) (Figure 4A,C,D). However, the starvation did not affect the expression of β-actin in myotubes (Figure 4A).

**Figure 5:**
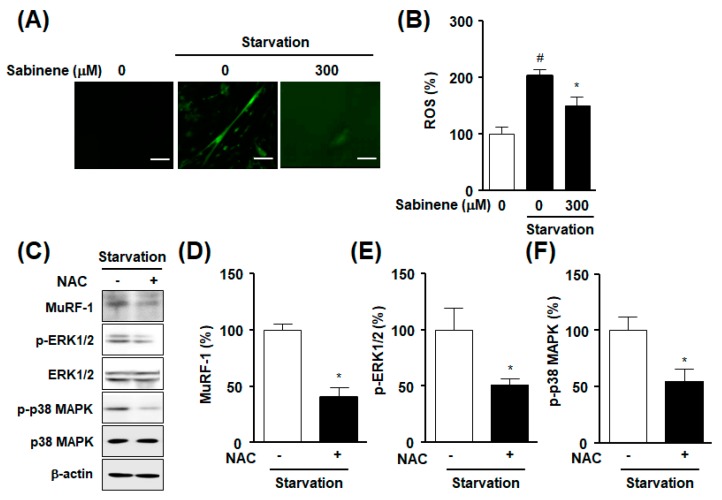
Effect of sabinene on the expression of reactive oxygen species in starved myotubes. (**A**,**B**) Effect of sabinene on the level of reactive oxygen species (ROS) in starved myotubes. L6 myotubes were incubated with serum-free DMEM in the absence or presence of sabinene (300 μM) for 18 h. Myotubes were loaded with fluorescent probe 2′,7′-dichlorodihydrofluorescein diacetate (H_2_DCFDA). The level of intercellular ROS was determined using a fluorescence microscope (**A**) and a microplate reader (**B**). (**A**) Representative images obtained by a fluorescence microscope. Green color indicates ROS positive response. *N* = 3, Scale bar: 100 μm. (**B**) Statistical graph of results measured by a microplate reader. The cell response in the quiescent state is expressed as 100% (*n* = 16). * *p* < 0.05 vs. starved myotubes in the absence of sabinene. # *p* < 0.05 vs. the quiescent state. (**C**–**F**) Effects of ROS inhibition on expression levels of MuRF-1 and MAPKs in starved myotubes. L6 myotubes were incubated in serum-free DMEM in the absence or presence of NAC (1 mM) for 18 h. These myotubes were immunoblotted with indicated antibodies. Panel (**C**) consists of representative images showing the altered expression of MuRF-1 and phosphorylation of MAPKs after treatment with *N*-acetyl-l-cysteine (NAC). The other panels (**D**–**F**) are statistical graphs obtained from panel (**C**). Expression of each protein in a NAC-untreated state is expressed as 100% (*n* = 4). * *p* < 0.05 vs. starved myotubes in the absence of NAC.

Previous studies have shown that ROS are associated with atrophy in starved myotubes [17] and that sabinene might have anti-oxidant activity to capture free radicals [24,25,26,27]. Thus, we next investigated the function of sabinene in ROS level in myotubes under starvation. Microscopic image analysis revealed that ROS production levels were upregulated in starved myotubes, whereas such upregulation was inhibited by sabinene at 300 μM (Figure 5A). Similar to this, fluorometric analysis results showed that 300 μM sabinene significantly attenuated the increase of ROS level in starved myotubes (Figure 5B). Furthermore, treatment with an ROS inhibitor *N*-acetyl-l-cysteine (NAC) (1 mM) decreased expression level of MuRF-1 and phosphorylation levels of ERK1/2 and p38MAPK in starved myotubes (Figure 5C–F). 

### 2.5. Effect of Sabinene on Fasting-Induced Gastrocnemius Muscle Atrophy 

**Figure 6:**
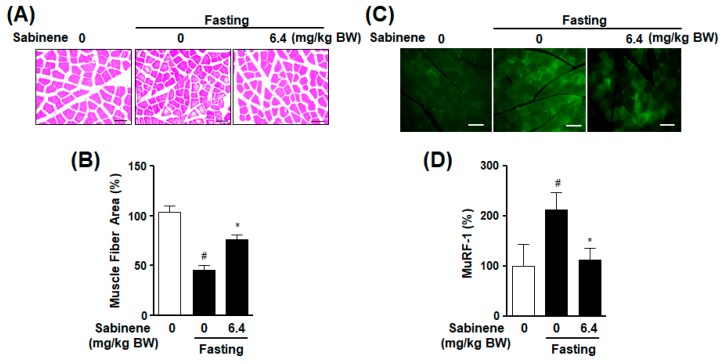
Effect of sabinene administration on fasting-induced gastrocnemius muscle atrophy in rats. Rats were orally treated with scoparone (6.4 mg kg/body weight (BW)) once every day for two days after fasting. Cross sections of gastrocnemius muscles isolated from each animal were stained with H and E or anti-MuRF-1 antibody. Muscle fiber size (area) and MuRF-1 expression were analyzed as described in Methods. (**A**) Representative image showing muscle fiber stained with Hematoxylin and Eosin (H and E). (**B**) Statistical graph obtained from panel (**A**). Muscle fiber area in gastrocnemius of non-fasted rats was considered as 100% (*n* = 10). Scale bar: 100 μm. * *p* < 0.05 vs. the fasted group in the absence of sabinene. # *p* < 0.05 vs. the non-fasted group. (**C**) Representative images showing MuRF-1 expression levels in muscles. (**D**) Statistical graph obtained from panel (**C**). MuRF-1 positive responses are expressed in light green color. Expression in non-fasted rat muscle was considered as 100% (*n* = 6). Scale bar: 100 μm. * *p* < 0.05 vs. the fasted group in the absence of sabinene. # *p* < 0.05 vs. the non-fasted group. BW: Body weight.

To evaluate the effect of sabinene on starvation-induced muscle atrophy, rats were orally treated with sabinene (6.4 mg/kg body weight) once daily during fasting for two days. Gastrocnemius muscle fiber areas were decreased by the fasting, whereas these decreases were reversed by the administration of sabinene (Figure 6A,B). Moreover, the administration of sabinene resulted in increases of gastrocnemius weights, which were decreased in fasted animals (data not shown). In addition, MuRF-1 expression levels were increased in gastrocnemius muscles of fasted animals, whereas these increases were decreased by the administration of sabinene (Figure 6C,D).

## 3. Discussion

Sabinene is a monoterpene isolated from EOs of many plants including medicine herbs [28,29,30]. Accumulated evidence indicates that the use of sabinene has potential as a therapy against a variety of diseases [31,32,33,34]. Sabinene has biological properties such as anti-fungal and anti-inflammatory activities [35,36]. In addition, it has been demonstrated that sabinene might have an antioxidant activity [24,25,26] and an anti-radical activity in relation to DPPH radicals [27]. Our previous investigations have revealed that sabinene is a component of CBMEO [20,22]. In the present study, we demonstrated for the first time that CBMEO attenuated size reduction of myoblasts under starvation state and that sabinene reversed the reduction in size of myotubes induced by starvation. Moreover, similar to the recovery effect of sabinene on muscle cell size, the reduction of fiber size of gastrocnemius muscle in fasted rats was prevented by sabinene administration. The loss of skeletal muscle mass can be caused by cachexia, malnutrition, denervation, bedding, and aging [37,38]. These findings imply that the development of new drugs using sabinene and CBMEO may be of great value for treating or preventing disorders related to skeletal muscle atrophy.

In the present study, sabinene attenuated the increase in the expression of MuRF-1 in both myotubes and gastrocnemius muscles under nutrient deprivation. MuRF-1 contains a RING-domain-related ubiquitination activity at its N-terminal end. It acts as an E3 ligase to regulate protein degradation and is a major factor that mediates the atrophy of a variety of cells, including skeletal muscle cells [3,8,12]. MuRF-1 and MAFbx are increased in atrophied muscles under various conditions [8,10,12,17]. Similarly, expression levels of MuRF-1 and MAFbx are increased in myoblasts and myotubes cultured in a serum-free medium [12,17]. Moreover, a fasting condition can induce the expression of proteins involved in protein degradation in the gastrocnemius muscle of mouse [39]. These results imply that sabinene may be able to control the development of skeletal muscle atrophy induced by malnutrition by inhibiting MuRF-1 ligase. 

It is well known that ROS are related to skeletal muscle damage and atrophy [17,40,41]. In the present study, we found that sabinene could inhibit ROS level elevated in starved myotubes. These findings indicate that sabinene may regulate muscle atrophy under fasting conditions via ROS-mediated signaling pathways. In a previous study, we demonstrated that overexpression of antioxidant protein DJ-1, a regulatory protein of oxidative stress, can diminish expressions of muscle atrophy markers MuRF-1 and MAFbx in undernutrition-induced atrophy of myoblasts [12]. Myotube atrophy is caused by increased ROS-induced MuRF-1 expression under starvation conditions, implying that increased an ROS level might cause atrophy of skeletal muscle under fasting conditions and that antioxidants might be able to prevent such atrophic effects on skeletal muscle [17]. In the present study, we also showed that ROS inhibitor NAC could downregulate the expression of MuRF-1 in starved myotubes. Therefore, ROS-mediated MuRF-1 signaling may be a potential event related to the regulation of fasting-induced muscle atrophy by sabinene treatment.

It has been reported that MAPKs such as p38 MAPK, ERK1/2, and JNK are molecules associated with muscle atrophy [11,23]. p38 MAPK and ERK1/2 can be activated by elevated intracellular ROS in skeletal muscle cells [13,41]. MuRF-1 expression can be inhibited by inhibitors of p38 MAPK and ERK1/2 in skeletal muscle cells [42,43]. These observations imply that ROS can act as upper signal molecules of the ERK1/2- or p38 MAPK-mediated MuRF-1 pathway. Previously, we have found that p38 MAPK activation is increased in both cast-immobilized gastrocnemius muscles and serum-starved L6 myoblasts and that a p38 MAPK inhibitor can attenuate the increase in expression of MuRF-1 in response to serum starvation [44]. These results strongly suggest that the expression of MuRF-1 in casted gastrocnemius muscle is mediated by the activation of ROS-mediated p38 MAPK. Along with p38 MAPK activation, ERK1/2 activation is also increased in atrophied skeletal muscles [23,45]. Increased level of phosphorylated ERK1/2 is associated with overexpression of both MAFbx and MuRF-1 ubiquitin ligases in C2C12 myotubes [46]. By contrast, inhibition of ERK1/2 activation can induce atrophy in C2C12 myotubes [47]. These results indicate that the role of ERK1/2 in skeletal muscle atrophy is controversial. In the present study, phosphorylation levels of ERK1/2 and p38 MAPK and expression levels of MuRF-1 were downregulated in starvation-induced atrophied myotubes after treatment with sabinene or ROS inhibitor. Moreover, sabinene decreased ROS generation and atrophy in myotubes under starvation. Our findings suggest that sabinene may play a role in preventing skeletal muscle atrophy via regulation of the ROS-mediated MAPK/MuRF-1 pathway. These findings could provide useful information in the development of therapeutic strategies.

In conclusion, the present study demonstrated that CBMEO inhibited starvation-induced atrophy in myoblasts and its bioactive component sabinene attenuated the atrophy of starved myotubes. MuRF-1 expression levels were increased in both starved myotubes and gastrocnemius muscles of fasted rats. Moreover, phosphorylation levels of p38 MAPK and ERK1/2 were enhanced in starved myotubes. These increases in MuRF-1 expression and phosphorylation of MAPKs were attenuated by treatment with sabinene. Sabinene inhibited ROS levels in starved myotubes. The ROS inhibitor reduced MuRF-1 expression and activations of MAPKs in starved myoblasts. In addition, oral administration of sabinene reversed the decreases of fiber sizes of gastrocnemius muscles in fasted rats. Collectively, our findings indicate that sabinene may be able to prevent muscle atrophy by inhibiting the ROS-mediated MAPK/MuRF-1 pathway. Therefore, sabinene and CBMEO may be promising agents with therapeutic potential for treating or preventing skeletal muscle atrophy.

## 4. Materials and Methods

### 4.1. Materials

Sabinene, NAC, and 4′,6-diamidine-2′-phenylindole dihydrochloride (DAPI) were purchased from Sigma–Aldrich (St. Louis, MO, USA). DMEM, FBS, horse serum, and phosphate-buffered saline (PBS) were purchased from Hyclone (Logan, UT, USA). Penicillin/streptomycin (P/S) and trypsin-ethylene diamine tetraacetic acid (EDTA) were purchased from Fisher Scientific (Pittsburgh, PA, USA). Antibodies including Alexa Fluor^®^ 488-conjugated goat anti-rabbit IgG (Life Technology, Carlsbad, CA, USA), anti-p38 MAPK, anti-phospho p38 MAPK, and anti-ERK1/2 (Cell Signaling, Danvers, MA, USA), anti-myosin heavy chain (MYH)-2, anti-phospho ERK1/2, anti-MuRF-1, and anti-β-actin antibodies (Santa Cruz Biotechnology, Santa Cruz, CA, USA) were used in this study. 

### 4.2. Animals and Muscle Atrophy

All animal experiments were performed in accordance with the Guide for the Care and Use of Laboratory Animals published by the US National Institutes of Health (NIH publication No. 85-23, revised 1996). They were approved by the Animal Subjects Committee following institutional guidelines of Konkuk University, Korea. Sprague-Dawley (SD) rats (8-week-old, male, 250–300 g; Nara Biotech, Seoul, Korea) were divided into three groups (*n* = 10 per group). One group of rats were orally administered sabinene once daily for 2 days (6.4 mg/kg body weight) and Tween-80 (1%) in saline solution. The other two groups of rats were treated with saline solution containing Tween-80 (1%) with or without food pellets. In fasted groups, food pellets were removed from cages of rats to be fasted. Two days later, rats were anesthetized intraperitoneally with Zoletil^®^ (40 mg/kg body weight; Virbac Laboratories, Carros, France) and Rompun^®^ (10 mg/kg body weight; Bayer Korea, Seoul, Korea). The adequacy of anesthesia was determined by the lack of reflex response to foot pinching in anesthetized rats. Gastrocnemius muscles of rats were dissected and isolated for analysis. 

### 4.3. Cell Culture and Cell Atrophy

L6 rat myoblast cells were purchased from the American Type Culture Collection (ATCC, Manassas, VA, USA). These cells were cultured in high glucose (4500 mg/L) DMEM supplemented with 10% FBS and 1% P/S in a humidified incubator at 37 °C with 95% air and 5% CO_2_. L6 myoblasts were grown to 70–80% confluence to induce differentiation into myotubes and incubated with a differentiation medium (2% horse serum in high-glucose DMEM) for 7 days. The differentiation medium was changed every 48 h. L6 myoblast-differentiating myotubes were incubated in a 24-well plate with serum-free DMEM (low glucose, 1000 mg/L) in the absence or presence of test agents for 18 h for each atrophy-related experiment. Myotubes were defined as all multinucleated cells positive for anti-MYH-2 and -DAPI antibodies. 

### 4.4. Extract of Chrysanthemum Boreale Makino Essential Oil

CBMEO was isolated as reported previously [22]. Briefly, CBM was cultivated in a practice farm of the Department of Cosmetic Science, Hoseo University, Asan, Korea, and identified by Dr. Jong-Cheol Yang, Division of Forest Biodiversity and Herbarium, Korea National Arboretum, Korea. A voucher specimen (CBMEO-0001) was deposited at the Herbarium of the College of Life and Health, Hoseo University. CBM flowers were harvested from CBM and air-dried for 24 h. The CBM was subjected to conventional steam distillation to extract EO. The yield of CBMEO was then confirmed. The obtained EO was stored at 4 °C in dark vials. The CBMEO was solubilized using HC-40, a non-cytotoxic solubilization agent for each experiment. 

### 4.5. Analysis of Essential Oils and Identification of Compounds

Compounds of CBMEO were identified and analyzed as reported previously [22]. Briefly, components were identified with a GC/MS and analyzed at the Korean Basic Science Institute (Seoul, Korea). GC/MS was performed with an Agilent 6890N GC/5975i MS instrument (Palo Alto, CA, USA), equipped with a DB5-MS capillary column (30 m × 250 μm, 0.25 μm film thickness). The carrier gas was helium with a flow rate of 1 mL/min. The injector port and interface temperatures were 280 and 300 °C, respectively. GC oven temperature was kept at 40 °C for 2 min, programmed to 230 °C at a rate of 5 °C/min, and then kept constant at 300 °C for 5 min. The split ratio was 1:10. Mass ranges were from 40 to 800 m/z. Retention indices (RIs) for all compounds were determined using the Kovats method with standard C7–C40 n-alkanes. Compounds were identified by comparing their RIs with Kovats indices and by matching their MS fragmentation patterns with the Wiley7NIST0.5L Mass Spectral library and catalogs of mass spectra.

### 4.6. Cell Viability Assay 

Cell viability was analyzed using XTT assay with a WelCountTM cell proliferation assay kit (Welgene, Daegu, Korea). Briefly, L6 myoblast cells (1 × 10^4^ cells/well) were seeded into a 96-well plate and incubated at 37 °C for 48 h with CBMEO. For myotube viability analysis, L6 myoblast cells (5 × 10^3^ cells/well) were seeded into a 96-well plate and incubated at 37 °C for 24 h with differentiation media for 7 days to induce myotubes. Differentiated myotubes were incubated at 37 °C for 48 h with sabinene. XTT (200 μg/mL) was added to each well and the plate was incubated for 2 h to allow the formation of the formazan dye. The absorbance was then measured at 450 nm using an Absorbance Microplate Reader (SpectraMAX^®^, Molecular Devices, CA, USA).

### 4.7. Immunoblotting

Cells were lysed with a buffer containing 1% NP40, 150 mM NaCl, 20 mM Tris–HCL (pH 7.5), 1 mM Na2EDTA, 1 mM EGTA, 1% sodium deoxycholate, 2.5 mM sodium pyrophosphate, 1 mM β-glycerophosphate, 1 mM Na3VO4, and 1 μg/mL leupeptin. Cell lysates containing proteins were subjected to sodium dodecyl sulfate polyacrylamide gel electrophoresis (SDS-PAGE) and transferred to polyvinylidene difluoride membranes. These membranes were blocked with PBS containing 5% bovine serum albumin (BSA), incubated at 4 °C overnight with primary antibodies (1:1000), and subsequently incubated with horseradish peroxidase-conjugated secondary antibody for 1 h at room temperature (RT). Protein bands were then visualized using enhanced chemiluminescence kits (Amersham Pharmacia, Piscataway, NJ, USA) and a luminescent image analyzer (LAS-4000, Fujifilm, Tokyo, Japan). Band intensities were quantified using the ImageJ software (NIH, Bethesda, MD, USA).

### 4.8. Measurement of Reactive Oxygen Species 

Intracellular ROS were detected with 2′,7′-dichlorodihydrofluorescein diacetate (H_2_DCFDA; Molecular Probes, OR, USA), according to the manufacturer’s instructions. Myotubes were differentiated in 24-well cell culture plates (SPL, Seoul, Korea) with L6 myoblasts (1 × 10^4^/well) for microscopic image analyses or in 96-well cell culture plates (SPL, Seoul, Korea) with L6 myoblasts (5 × 10^3^/well) for fluorometric analyses. Differentiated myotubes were cultivated in serum-free DMEM with or without test agents for 18 h and stained with 5 μM H2DCFDA for 30 min in a humidified incubator at 37 °C with 95% air and 5% CO_2_. Image analyses were performed with stained cell images taken by an inverted fluorescence microscope (Axio200, Carl Zeiss, Oberkochen, Germany). Fluorometric analyses were done using an Absorbance Microplate Reader (SpectraMAX^®^, Molecular Devices, CA, USA) at an excitation wavelength of 488 nm and an emission wavelength of 519 nm.

### 4.9. Cell Morphologic Analyses 

For morphometric analyses of myotubes, L6 myotubes were fixed with 4% paraformaldehyde at 4 °C overnight. After washing with PBS, cells were blocked in PBS containing 0.1% tween 20 and 3% BSA for 1 h at 4 °C. These myotubes were then incubated with primary antibody solution of anti-MYH-2 (1:100) overnight at 4 °C. After washing three times, myotubes were incubated with Alexa Fluor 488-conjugated secondary antibody (1:500) at RT for 1 h, followed by treatment with DAPI for 20 min at RT. Under each experimental condition, images were taken at five fields in a random fashion with an upright fluorescence microscope (BX61-32FDIC, Olympus). Every myotube from these images was then measured with ImageJ software to determine its diameter. Diameters of individual myotubes were calculated as an average diameter of five points along the length of the myotube in each image. This analysis was repeated for 12 independent experiments per group. Results are expressed as percentages relative to the control group value.

To measure myoblast size, fixed L6 myoblasts were stained with crystal violet (Sigma–Aldrich). Images for each experimental condition were captured at five fields in a random fashion with a BX51 light microscope (Olympus, Tokyo, Japan). Each myoblast from these images was then measured for its area using ImageJ. The size of each myoblast was calculated as an average area of total myoblasts in each image. The analysis was repeated in 9 or 12 independent experiments per group. Results are expressed as percentages relative to the control group value.

### 4.10. Histochemical and Immunohistochemical Analyses

Muscle morphological analysis was performed for gastrocnemius muscles isolated from rats. Briefly, these isolated muscles were washed with ice-cold PBS, fixed with 4% paraformaldehyde, segmented, and embedded in paraffin. These segments were cross-sectioned (7 μm in thickness), cleared with xylene, and hydrated with ethanol. These sections were then stained with Hematoxylin and Eosin (H and E). Images were captured in a random fashion under a Nikon Eclipse Ni-U upright microscope (Nikon, Tokyo, Japan). The cross-sectional area of the muscle fiber was measured for all muscle fibers of 5 images selected from each animal. The average area of muscle fibers was calculated as the mean area value of total muscle fibers in 5 images. Ten animals per experiment group were used for muscle fiber area analysis. Results are expressed as percentages relative to the control group value.

For analysis of MuRF-1 expression in isolated gastrocnemius muscles, some sections were cleared with xylene and hydrated with ethanol, followed by antigen retrieval with 0.01 M sodium citrate (pH 7.4) for 5 min at 4 °C. They were then kept at RT for 30 min. After these sections were stained with anti-MuRF-1 antibody (1:100) and Alexa Fluor 488-conjugated rabbit IgG antibody (1:500), images were captured under a BX61-32FDIC. The intensity of MuRF-1 was measured using a Metamorph imaging software (Molecular Devices). 

### 4.11. Statistical Analysis

All experimental results are expressed as mean ± standard error of the mean (S.E.M.). Statistical evaluation was performed by one-way analysis of variance (ANOVA), followed by a Bonferroni’s post hoc test for multiple comparisons and by unpaired Student′s *t*-test for comparisons between pairs of groups. All data were analyzed using GraphPad Prism version 5.0 (San Diego, CA, USA). Data were regarded as having significant difference when the P value was less than 0.05.

## Abbreviations

ERK	Extracellular signal-regulated kinase
CBMEO	*Chrysanthemum boreale* Makino essential oil
DAPI	4′,6-Diamidine-2′-phenylindole dihydrochloride
DMEM	Dulbecco’s modified Eagle’s medium
FBS	Fetal bovine serum
GC/MS	Gas chromatography/mass spectrometry
H_2_DCFDA	2′,7′-Dichlorodihydrofluorescein diacetate
MAFbx	Muscle atrophy F-Box
MAPK	Mitogen-activated protein kinase
MuRF-1	E3 Ubiquitin ligase muscle ring-finger protein-1
MYH	Myosin heavy chain
NAC	*N*-acetyl-l-cysteine
ROS	Reactive oxygen species
PBS	Phosphate buffered saline
P/S	Penicillin/streptomycin
RT	Room temperature
SD	Sprague-Dawley
XTT	2,3-Bis (2-methoxy-4-nitro-5-sulfophenyl)-2H-tetrazolium-5-carboxanilide inner salt
